# Improving the thermostability of alpha-amylase by combinatorial coevolving-site saturation mutagenesis

**DOI:** 10.1186/1471-2105-13-263

**Published:** 2012-10-11

**Authors:** Chenghua Wang, Ribo Huang, Bingfang He, Qishi Du

**Affiliations:** 1 Biotechnology and Pharmaceutical Engineering, Nanjing University of Technology, Xinmofan Road 5, Nanjing, 210009, Jiangsu, China; 2 State Key Laboratory of Enzyme Technology, National Engineering Research Center for Non-food Biorefinery, Guangxi Key Laboratory of Biorefinery, Guangxi Academy of Sciences, Nanning, Guangxi, 530007, China; 3 College of Life Science and Technology, Guangxi University, Nanning, Guangxi, 530004, China

## Abstract

**Background:**

The generation of focused mutant libraries at hotspot residues is an important strategy in directed protein evolution. Existing methods, such as combinatorial active site testing and residual coupling analysis, depend primarily on the evolutionary conserved information to find the hotspot residues. Hardly any attention has been paid to another important functional and structural determinants, the functionally correlated variation information--coevolution.

**Results:**

In this paper, we suggest a new method, named combinatorial coevolving-site saturation mutagenesis (CCSM), in which the functionally correlated variation sites of proteins are chosen as the hotspot sites to construct focused mutant libraries. The CCSM approach was used to improve the thermal stability of α-amylase from *Bacillus subtilis* CN7 (Amy7C). The results indicate that the CCSM can identify novel beneficial mutation sites, and enhance the thermal stability of wild-type Amy7C by 8°C ( $T_{50}^{30}$), which could not be achieved with the ordinarily rational introduction of single or a double point mutation.

**Conclusions:**

Our method is able to produce more thermostable mutant α-amylases with novel beneficial mutations at new sites. It is also verified that the coevolving sites can be used as the hotspots to construct focused mutant libraries in protein engineering. This study throws new light on the active researches of the molecular coevolution.

## Background

Directed protein evolution is invaluable in engineering biocatalysts for better properties, such as enhancements in activity, stability, and enzyme selectivity [1,2]. However, it is usually limited by its inability to generate high-quality mutant libraries containing more beneficial variants. This is especially problematic considering the combinatorial complexity of mutant libraries and the huge sequence space [3,4]. Constructing focused mutant libraries at defined hotspot residues is considered to be one of the most promising ways of improving directed protein evolution [3-5]. Much of pioneering work has been complemented by Reetz’s team [6-8].

All existing focused mutant library methods can be essentially classified into two categories: structure-based approaches and sequence-based approaches. The former includes combinatorial active site testing (CAST), B-factors, and knowledge-based potential analysis [6-10]. The latter includes protein design automation (PDA) [11], residual coupling analysis (RCA) [12], and ConSurf [13]. While the aforementioned methods depend primarily on the evolutionary conservation information to find out the hotspot residues, there are some other important functional and structural determinants desirable to be taken into consideration, such as the functionally correlated variation information--coevolution.

Co-evolution is the correlated variation of protein sites promoted by selective pressures [14]. The cooperation between residues at the coevolving sites, which usually takes the form of compensatory interactions, synergistic effects, allosteric interactions, and epistatic interactions [15-19], determines the structure and function of proteins [20,21]. In recent years much attention has been paid to find the coevolving residues, as well as the reasons why residues co-evolve [14,21-28], but few experimental design methods based on the coevolution and successful examples of using them have been reported.

In this study, we propose a method, combinatorial coevolving-site saturation mutagenesis (CCSM), which chooses the coevolving sites of proteins as hotspot residues to construct focused mutant libraries. We also describe the successful use of the CCSM method to improve the thermostability of α-amylase.

## Results and discussion

α-Amylase is an important industrial biocatalyst in starch liquefaction processes and a valuable model enzyme for studies of thermal adaptation in proteins [29]. We used the CCSM approach to improve the thermostability of α-amylase (Amy7C) to demonstrate the feasibility of this method.

### Spotting the coevolving sites in Amy7C

**Figure 1:**
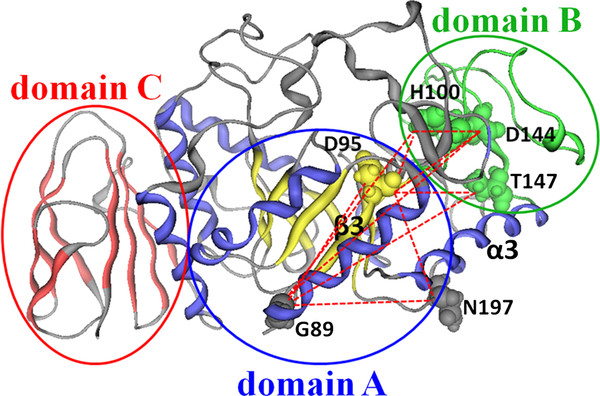
**Distribution of co-evolving sites in Amy7C.** Amy7C is shown in cartoon form, and the coevolving sites are shown in filled balls. The catalytic domain A, consisting of a closed eight-stranded parallel β sheet barrel (yellow) surrounded by eight α helices (blue), is circled in blue. Domain B that protrudes between third β-strand and third α-helix of domain A, is circled in green. The C-terminal β-sheet (red), domain C is circled in red. The sequences linking the domains are shown in gray. The dashed red line indicates the co-evolutionary relationship between each pair of co-evolving sites.

Six coevolving residues and 10 pairs of co-evolutionary interactions were identified in Amy7C during step 1 of the CCSM approach (see Additional file 1, Additional file 2: Table SA2 and Additional file 3: Table SA3 for computational details). As shown in Figure 1, among the six residues, H100, D144, and T147 are located in domain B, and G89, D95 and N197 are in domain A. In domain A, the G89 is at the loop linking α2 and β3, D95 is on β3, and N197 is at the loop linking α4 and β5. Except for D95, all the coevolving sites are situated exactly in the so-called “stability face” of Amy7C. This stability face includes domain B and the loops linking the α helices with the subsequent β strands of TIM barrel of domain A [30]. The above observation of coevolving sites is consistent with previously published works, which demonstrated the thermostable mutations concentrated on the stability face, by conventional blind or rational protein engineering experiments [31]. However, the coevolving sites of Amy7C spotted by us in this work are distributed across a larger region than the stability face defined by other authors [30], and they are different sites from those identified by other authors [31].

The average distance between all coevolving sites in Amy7C is in the range 17.3 ± 7.31 Å, which is much greater than that reported by other research teams [26,32]. The distance between coevolving sites are significantly greater than the distance used to define hotspot sites in previous studies, which is usually about 5 Å [6,11]. The differences between the coevolving sites in this study and the hotspot sites found by previous studies must be attributed to the prediction methods, because the previous studies identified hotspots by evolutionary conservative information-based methods, such as the sequence alignment-based method and distance-based method [6-8], which could not usually find the coevolving sites located as distant as >17 Å apart.

### Construction and screening of CCSM libraries

Ten CCSM libraries were constructed at coevolving-sites and explored using the HTS method, which is based on the starch-iodine method and DNS method [33,34] (see Additional file 1 for details). All possible combinations and permutations of amino acid residues are explored in the CCSM library through simultaneous and random mutation of the coevolving-sites using the NNK(G/T) degenerate primers (see Table SA1 in Additional file 4: Table SA1).

A total of 10,010 clones were randomly selected and screened using the starch-iodine method in the first screening. The majority of the variants displayed impaired activities, and only about 10% retained any obvious starch hydrolytic ability relative to parental Amy7C. The active variants made up less than 5% of the three libraries of G89H100, G89D144, and G89T147. Active variants of the other seven libraries made up around 12.5%.

**Figure 2:**
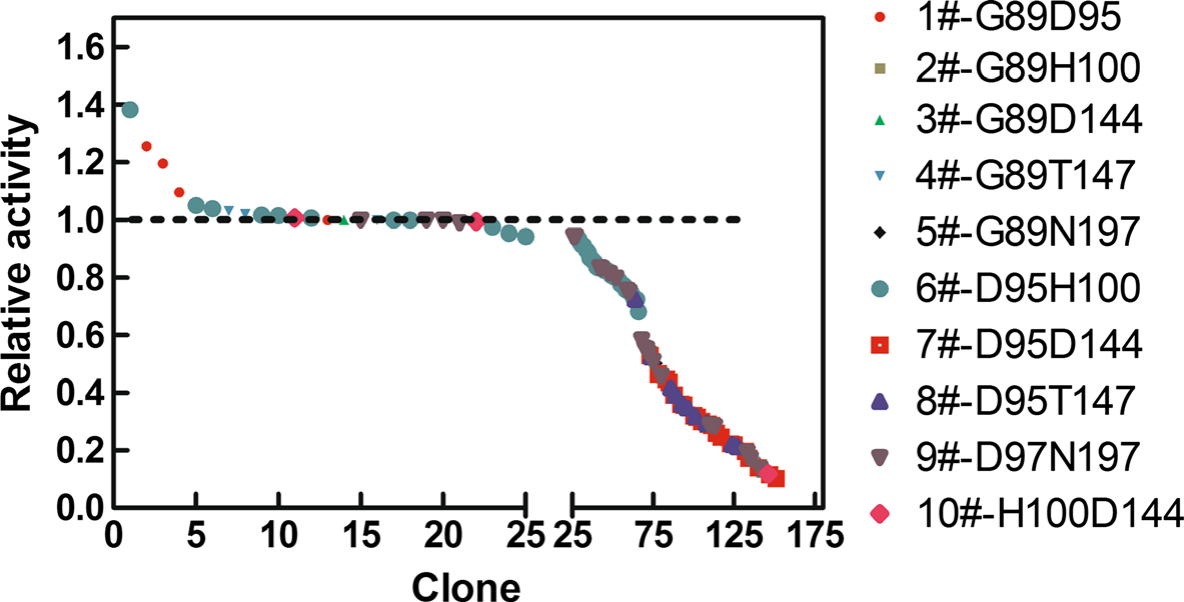
**Activity landscape of the CCSM libraries plotted in descending order.** The dotted line indicates relative activity of the wild type amylase, which is scaled to 1. The top 25 variants are shown in the first segment of the horizontal axis, the other 127 variants are shown in the second segment.

A total of 880 potential hits in the initial screening were rescreened by the DNS method using freshly transformed cells to discard false positives. In the 880 variants, 152 variants showed above 10% of the parent enzyme’s activity, and only 76 variants displayed more than 50% activities. The activity landscape of the top 152 variants is shown in Figure 2. It can be seen that the top 25 variants, as shown around the dotted line in the first segment of the horizontal axis in Figure 2, mainly came from D95H100, G89D95 and D97N197 libraries, while variants from the H100D144, D95T147 and D95D144 libraries showed relatively low activities.

### Rescreening of CCSM libraries

**Figure 3:**
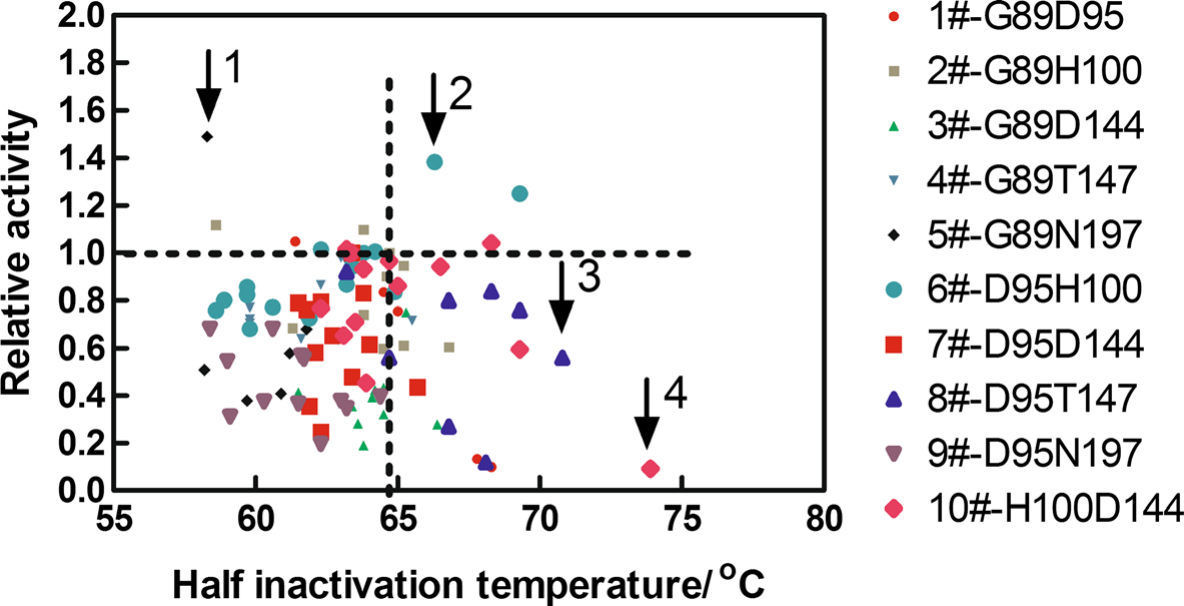
**The relative activity and**$T_{50}^{30}$**value distribution of variants in the CCSM libraries.** Relative activities (vertical axis) of variants are plotted versus $T_{50}^{30}$values (horizontal axis). The four representative variants N197C, H100I, T147P, and H100MD144R are marked out by “1”, “2”, “3”, and “4”, respectively. The horizontal dotted line represents the relative activity of the wild type amylase, and the vertical one denotes its half inactivation temperature ( $T_{50}^{30}$).

The top 120 variants (12 variants in each CCSM library) were rescreened by characterizing their relative activities and their $T_{50}^{30}$values compared to the wild type, using freshly prepared crude enzymes. The average relative activity of the 120 variants was found to be 0.68 ± 0.28, in contrast to the 1.02 ± 0.22 of wild-type enzyme. The average $T_{50}^{30}$value of the 120 variants was found to be 63.5 ± 2.86°C, compared to 64.8 ± 1.04°C of the wild-type enzyme. A total of 98 variants had half-inactivating temperature above 58°C and retained more than 10% relative activity in comparison to the wild-type enzyme. Figure 3 depicts the relative activity and $T_{50}^{30}$values of the top 98 in the 120 variants. From the Figure 3, we can see that among the 24 most thermostable variants compared with the wild type, 16 contained one of the H100, D144 and T147 sites, so it appears that these three sites in domain B are primarily responsible for the most thermally stable variants.

### Sequence analysis of the CCSM mutants

The sequence analysis of top 98 variants in the rescreening indicated that 28 variants (28.6%) had not changed at all (could be regarded as false positive), 35 variants (35.7%) had mutated at single sites, and 35 variants (35.7%) had double mutations at the designed co-evolving sites. Table 1 summarizes the amino acids and codons distributed over each site. Most of the mutations observed in our CCSM library require a minimum of two nucleotide changes per codon, and some can only be by more than three nucleotide changes (Table 1). These nucleotide changes are nearly inaccessible for conventional error-prone PCR and single-gene DNA shuffling methods [11].

**Table 1:** The distribution of amino acids and codons at the 6 coevolving sites in the sequenced 92 amylase variants

**Amino**	**Positions**						
**Acid**	**G89**	**D95**	**H100**	**D144**	**T147**	**N197**	**No.**
W. T.	GGC	GAT	CAT	GAT	ACT	AAT	
Ala (A)	G**CG**_2_					**CGC**_1_	3
Cys (C)	**TTT**_2_				**TG**T_3_	**TG**T_7_	12
Asp (D)	**CTG**_1_					**G**AT_1_	2
Glu (E)	G**AG**_1_,			GA**G**_3_			4
Phe (F)	**TTT**_1_						1
Gly (G)	GG**G**_5_,GG**T**_3_			**GGG**_4_,G**G**T_4_		**GGG**_3_	27
His (H)		**C**AT_1_					1
Ile (I)			**AT**T_2_				2
Lys (K)						AA**G**_1_	1
Leu (L)	**CTG**_1_						1
Met (M)			**ATG**_4_			A**TG**_2_	6
Asn (N)	**AAT**_1_		**A**AT_2_		A**A**T_3_		6
Pro (P)					**C**CT_3_		3
Gln (Q)	**CAG**_1_		CA**G**_1_			**C**A**G**_1_	3
Arg (R)	**C**G**T**_3_,**C**GC_3_	**CGG**_1_	C**GG**_1_,C**G**T_3_	**CGG**_1_	**CG**T_4_,**CGC**_3_	A**GG**_1_	20
Ser (S)			**AG**T_1_		**T**CT_1_	**TCG**_5_, A**G**T_1_	3
Thr (T)	**ACA**_1_						1
Val (V)				G**T**T_3_	**GTG**_3_		6
Trp (W)				**TGG**_1_			1
Tyr (Y)							0
Type	11	2	6	5	6	9	

Subscript numbers represent the frequencies of the codons in the 92 sequenced variants. Underlined bold letters indicate substitution to the wild type nucleotide.

No. = total number of codons different from the wild type for each amino acid. Type = type number of amino acids appeared at each site. W. T. = wild type.

All the coevolving sites showed dramatic variation in either single or double mutations, except D95 showed only two double mutations, i.e., D95HT147S (CATTCT) and G89FD95R (TTTCGG). G89 and N197 were found to be the most diverse mutation sites, which displayed 11 and 9 different kinds of amino acids respectively (Table 1). Previous studies have shown that the eight strands and eight helixes of the TIM barrel of domain A are vital to the stability of the structure [35,36], and few beneficial mutations can exist there. In this study, both D95HT147S and G89FD95R were found to involve changes to the residue D95 of the β3 in the TIM barrel of the domain A. The detrimental effects caused by D95 site mutation must be compensated by the covariation at the other coevolving site, like the T147S in D95HT147S. The similar but beneficial cooperation may also take place between coevolving residues in improved variants. The positions and interactions between coevolving residues in some example variants are shown in Figure A1 (see Additional file 5: Figure SA1).

The aforementioned “false positive” phenomenon of high percentages of same sense mutations (28.6%) and single mutants (35.7%) upon rescreening should probably be attributed to the relatively lenient criteria adopted in our library construction and screening procedures. NNK degeneracy in the primers offers a variety of 32 codons and encodes all possible 20 amino acids, so it will inevitably produce same sense mutations in the library construction. Meanwhile, the selection criteria for the sequenced 98 variants were set at above 58°C and at more than 10% residual relative activity, which are far below that (about 64.8°C and 50%) of the wild-type enzyme (Figure 3).

### Validation of the representative improved variants

To evaluate the effects of CCSM in improving the thermal stability of Amy7C, the wild-type Amy7C and four representative variants of N197C, H100I, T147P and H100MD144R (denoted by “1”, “2”, “3” and “4” in Figure 3), were purified to homogeneity and characterized [see Addition file 1. There appeared to be a tradeoff between thermal stability and catalytic activity of Amy7C variants [37]. Amy7C showed a $k_{\mathrm{cat}}$value of 1260.55 s^-1^ and a $T_{50}^{30}$value of 62.3°C. N197C showed a reduced $T_{50}^{30}$value of 58.3°C and a slightly higher catalytic activity $k_{\mathrm{cat}}$value of 1298.37 s^-1^. From the H100I, to T147P, to H100MD144R, the $T_{50}^{30}$values increased by 4.5°C, 7°C, and 8°C, while the catalytic activities range from 1.04-fold, to 0.74-fold, to 0.31-fold, respectively.

Due to both the academic and industrial values, amylase has been extensively studied in different laboratories, and numerous engineering work has been done to increase the thermostabiliy [38]. Among the most excellent works, Machius et al. have successfully identified some beneficial amino acid substitutions in an amylase BLA from *Bacillus licheniformis *[39-44], and even created a hyperthermostable variant with 23°C higher than the wild-type enzyme by substituting 7 amino acids [31,38,44]. However, to the best of our knowledge, if the test conditions and sources of α-amylases are not considered, the $T_{50}^{30}$ increase of 8°C observed in this study is the largest ever achieved with a single round by introducing up to two point mutations into wild-type α-amylases [31].

As a coevolving strategy, our method also identified stabilizing variants with only single mutations at certain coevolving sites, such as H100I and T147P mutations (see above). From time to time, there is no difference between our coevolving method and traditional mutation methods such as error-prone PCR and DNA shuffling in generation of stabilizing single mutations, but in fact our single mutation should be regarded as same as other coevolving double mutations since the newly introduced single amino acid has somehow improved the coordination between two residues at the coevolving sites, and made them perfectly match in certain performances such as thermostability.

So, the above validation results clearly indicate that the screened beneficial variants changed at the coevolving sites, and the new amino acid combinations and the cooperation between them at coevolving sites brought greater thermal stability than the wild-type enzyme. It also indicates that CSSM may be more effective in generating desired mutations because it involves at least two coevolving sites that may be located in some far-away positions in protein sequences but more likely in the proximity to each other on the three dimensional structure of the proteins, and since it involves the coordinate changes in both amino acid positions they will then be more likely to co-evolve towards some direction we desired, which could be imagined as coordinated “directed evolution”, in sharp contrast to the ordinary “directed evolution”. The method proposed here only uses the protein sequence to detect coevolving sites, then employs combinatorial saturation mutagenesis to create mutations changing at both coevolving sites, and then screens out beneficial variants. So, it seems promising that the CSSM method should be applicable to many interesting enzymes other than α-amylase.

## Conclusions

This study shows that the new method of choosing the coevolving sites as the hotspots for constructing focused mutant libraries leads to improved variants with novel beneficial mutations at new sites. The successful application of CCSM in improving the thermostability of α-amylase in this study also throws new light on the active researches of the molecular coevolution.

## Methods

The CCSM approach combines coevolving site identification with combinatorial saturation mutagenesis [45] and high throughput screening method. The CCSM approach is carried out in three steps.

### Step 1: Identification of coevolving sites

The coevolving sites in protein families and the coevolving pairs of residues in a query protein sequence are predicted by carrying out the following five methods successively, according to the state-of-the-art row and column weighting of mutual information (RCW-MI) method [22]. Firstly, the query sequence is compared to the Uniprot [46] by BLASTP, and the compatible homologous sequences are retrieved. Secondly, the homologous sequences are aligned via the MAFFT [47] method to build the family sequence alignment. Thirdly, the alignment is then processed by MaxAlign [48] to diminish the number of gapped columns in the alignment. Fourthly, the mutual information between each two sites is calculated by the equation (1) [22], which consists of the mutual information matrix.

(1) $$MI\left(A:B\right)=\sum_{i}\sum_{j}P\left(a_{i},b_{j}\right)\log_{20}\left[\frac{P\left(a_{i},b_{j}\right)}{P\left(a_{i}\right)P\left(b_{j}\right)}\right]$$

Where, *MI(A:B)* is the mutual information between two sites *A* and *B*, and i and j run through all the occurring amino acids in each site. The base 20 for the logarithm is the number of letters in the protein alphabet. *P(a*_*i*_*)*, *P(b*_*j*_*)* and *P(a*_*i,*_*b*_*j*_*)* are the observed frequencies of amino acids *a*_*i,*_, *b*_*j*_ and *(a*_*i,*_, *b*_*j*_), respectively.

Fifthly, each site pair of the mutual information matrix is weighted by the average score of constituting sites according to according to the equation (2) [49].

(2) $$RCW\left(A:B\right)=\frac{MI_{\mathrm{ij}}}{\left(MI_{i.}+MI_{.j}-2MI_{\mathrm{ij}}\right)/\left(n-1\right)}$$

Where, *RCW(A:B)* is the row and column weighted mutual information between *A* and *B* sites, *MI*_*ij*_ represents the mutual information between sites *i* and *j*, *MI*_*i.*_ stands for the summation over all sites in row *i*, *MI*_*.j*_ denotes the sum of the Mutual Information matrix over all lines in column *j*, *n* is the number of alignment sequences.

The coevolving sites prediction in this research was carried out by the above method via InterMap3D server [50], which is an available server to the general community for predicting and visualizing co-evolving proteins residues.

### Step 2: Construction of combinatorial saturation mutagenesis library at coevolving sites

The CCSM libraries are constructed by simultaneously and randomly mutating the coevolving sites using the protocol of QuickChange® XL Site-Directed Mutagenesis Kit from Stratagene (La Jolla, CA) [51]. Complementary primers 33–35 nucleotides in length, which include NNK (G/T) degenerate codons exactly matching the coevolving sites, were designed. For each pair of coevolving sites, PCR reactions were performed using two pairs of complementary primers, each pair corresponding to a coevolving site. After removal of the methylated template plasmid with DpnI enzyme, PCR products were transformed into *E. coli* XL1-Blue competent cells by chemical transformation [52]. The transformed cells harboring the CCSM libraries were plated on LB agar supplemented with antibiotics.

### Step 3: Screening of the improved mutants

We used high throughput screening method to identify improved mutants from the CCSM library in a statistically significant way. In this study, mutant enzymes are assayed for residual activity relative to the wild-type strain after heat treatment and assayed for thermo-stability with respect to the half-inactivation temperature ( $T_{50}^{30}$). Clones demonstrating the highest thermostability and survival relative activity are rescreened, and the genes of rescreened variants are sequenced to identify the mutations. The identified mutant enzymes are purified, and the $T_{50}^{30}$value and catalytic activity are further characterized to confirm the initial screening results.

The α-amylase Amy7C [GenBank: JN980090], derived from *Bacillus subtilis* CN7, was used to demonstrate the utility of our CCSM method. Amy7C is Ca^2+^-independent and is relatively stable at a wide range of pH values. However, its thermostability is not sufficient for use in starch simultaneous saccharification and liquefaction processes. *Bacillus subtilis* CN7 was screened and deposited in our laboratory. The plate plasmid pSA7C, the host strains *E. coli* XL1-Blue and *E. coli* JM109, and nucleotide primers are listed in Table A1 (see Additional file 4: Table SA1). The primers were synthesized by Generay (Shanghai, China) and gene sequencing was performed by Shanghai DNA Biotechnologies (Shanghai, China). All the detailed materials and methods can be found in supporting materials (Additional file 1).

## Abbreviations

CCSM: Combinatorial Coevolving-site Saturation Mutagenesis; RCW-MI: Row and Column Weighting of Mutual Information; HTS: High-Throughput Screening.

## Competing interests

The authors declare that they have no competing interests.

## Authors’ contributions

CHW participated in the design of the study, carried out the molecular manipulations, interpreted the results and wrote the manuscript. RBH conceived of the study, performed data analysis and wrote the manuscript. QSD participated in data analysis and helped CHW to revise the manuscript. BFH helped CHW to implement the study and provided the guidance. All authors read and approved the final manuscript.

## Supplementary Material

Additional file 1 **Detailed experimental procedure for improving the thermostability of AmyC using CCSM.** This file provides the experimental details suitable for applying the CCSM approach to the improvement of the thermostability of Amy7C. It includes the materials and methods, and the methods part includes selection of coevolving residues and protein modelling, construction of CCSM libraries at coevolving sites, expression of mutant α-amylases and preparation of crude enzymes, initial screening of CCSM library by starch-iodine method, rescreening of potential hits by DNS, and purification and characterization of α-amylase. Click here for file

Additional file 2 **Table SA2.** Information on sequences homologous to Amy7C identified in Uniprot. This file provides the information including Accession number, name, origin strain and number of amino acids on the sequences analogous to Amy7C identified in Uniprot and employed to find the coevolving sites through the InterMap3D server in this study. Click here for file

Additional file 3 **Table SA3.** Multiple Sequence Alignment in CLUSTAL format obtained by MAFFT method in this study. This file provides the Multiple Sequence Alignment in CLUSTAL format of the sequences homologous to Amy7C obtained by MAFFT method to find the coevolving sites through InterMap3D server in this study, Click here for file

Additional file 4 **Table SA1.** Plasmids, strains, and primers used in this study. This file includes the plasmids, host strains, and nucleotide primers used in this study. Click here for file

Additional file 5 **Figure SA1.** Close-up views of residues at coevolving sites in Amy7C and its variants. This file depicts the position and interaction of the residues at coevolving sites in Amy7C (A), H100I (B), D95HT147S (C), H100MD144R (D), T147P (E), N197C (F), and G89FD95R (G). Click here for file
