# Supplementary material for: Improving the thermostability of alpha-amylase by combinatorial coevolving-site saturation mutagenesis
Source: BMC Bioinformatics. 2012 Oct 11;13:263. doi: 10.1186/1471-2105-13-263 (PMC3478181; doi:10.1186/1471-2105-13-263)
Supplement: Additional file 1 — Detailed experimental procedure for improving the thermostability of AmyC using CCSM. This file provides the experimental details suitable for applying the CCSM approach to the improvement of the thermostability of Amy7C. It includes the materials and methods, and the methods part includes selection of coevolving residues and protein modelling, construction of CCSM libraries at coevolving sites, expression of mutant α-amylases and preparation of crude enzymes, initial screening of CCSM library by starch-iodine method, rescreening of potential hits by DNS, and purification and characterization of α-amylase. [file 1471-2105-13-263-S1.docx]

**Detailed experimental procedure for improving the thermostability of AmyC using CCSM.**

**Chenghua Wang^1,2^, Ribo Huang^1,2,3^**^*^**, Bingfang He^1^, Qishi Du^2,3^**

^1^ Biotechnology and Pharmaceutical Engineering, Nanjing University of Technology, Xinmofan Road 5, 210009 Nanjing, Jiangsu, China. ^2^ State Key Laboratory of Non-Food Biomass and Enzyme Technology, National Engineering Research Center for Non-food Biorefinery, Guangxi Academy of Sciences, Guangxi Key Laboratory of Biorefinery, 530007 Nanning, Guangxi, China. ^3^ College of Life Science and Technology, Guangxi University, 530004 Nanning, Guangxi, China

Email: CHW: [st.chwang@gmail.com](mailto:st.chwang@gmail.com); RBH: [rbhuang@gxas.ac.cn](mailto:rbhuang@gxas.ac.cn); BFH: [hebingfang@njut.edu.cn](mailto:hebingfang@njut.edu.cn);

QSD: [qishi_du@yahoo.com.cn](mailto:qishi_du@yahoo.com.cn)

^*^Corresponding author

This file provides experimental details suitable for applying the CCSM approach to the improvement of the thermostability of Amy7C. It contains two parts of materials and methods.

**Materials**

All the conventional chemicals are from commercial sources with no less than reagent-grade purity. The soluble starch (from potatoes) was purchased from Sinopharm Chemical Reagent Co., Ltd (Shanghai, China). PrimerSTAR® DNA polymerase, dNTPs were from TAKARA (Japan). Dpn I endonuclease with 10X buffer were from New England Biolabs (Shanghai, China). The Bradford protein assay kit was from Generay (Shanghai, China). The plasmid pSA7C, the host strain *E. coli* XL1-Blue and nucleotide primers are listed in Table A1 (See Additional file 2). The primers were synthesized by Generay (Shanghai, China) and gene sequencing was performed by Shanghai DNA Biotechnologies (Shanghai, China).

**Methods**

This section describes the experimental details of applying the CCSM approach to the improvement of the thermostability of Amy7C step by step.

***Selection of coevolving residues and protein modeling***

The coevolving residues of Amy7C are predicted by the state-of-the-art row and column weighting of mutual information (RCW-MI) method [1] via the InterMap3D server [2,3]. The default parameter values were used except for the following settings: 25% of minimum length of the query protein and MAFFT method were set for the homology alignment, and RCW-MI method and up to 3 residues in the coevolving pairs were set for co-evolution analysis. Under the condition of the above parameter settings, 16 representative sequences homologous to Amy7C were identified in Uniprot and employed to find the coevolving sites by the InterMap3D server in this study, the information of these homologous sequences can be found in Table A2 (see Additional file 3), and the Multiple Sequence Alignment in Clustal format by MAFFT method can be found in Table A3 (see Additional file 4). Because the Amy7C shows the highest homology (90% identity) to the α-amylase from Bacillus subtilis [PDB: 1BAG], one homologous model was constructed via the Swiss-Model Protein Modeling server [4,5]. The chosen coevolving residues were depicted on the computational model of Amy7C with Pymol 0.99 [6].

***Construction of CCSM libraries at coevolving sites***

The CCSM libraries were constructed by simultaneously and randomly mutating the coevolving sites using the QuickChange® XL Site-Directed Mutagenesis Kit from Stratagene (La Jolla, CA) [7]. According to the manufacturer’s instructions, complementary primers 33–35 nucleotides in length, including NNK (G/T) degenerate codons exactly matching the coevolving sites, were designed. The primer names were designated using the names of the mutated residues (single letter) + site numbering + SM (short for saturation mutagenesis) + “-” + primer orientation (S-sense primer, A-antisense primer). The primers were listed in Table A1 (see Additional file 2).

For each pair of coevolving sites, PCR reactions were performed in a final volume of 50 μl containing 0.25 μM of each primer (two pairs of complementary primers, each pair corresponds to a coevolving site), 10 ng of template plasmid (pSA7C), dNTPs (0.25 mM each),1x PrimeSTAR buffer (Mg2+ plus), and 2 units of PrimeSTAR HS DNA polymerase. PCR conditions were as follows: 95 ºC for 3 min (1 cycle), 98 ºC for 15 s, 68ºC for 5 min 40 s (30 cycles), and 72 ºC for 10 min (1 cycle). PCR products were monitored by electrophoresis using a 1% agarose gel in TAE buffer. The certified PCR products were digested with two successive additions of Dpn I (1U/20ul) to remove the methylated template plasmid. Each digestion was performed at 37 °C for 2 hours. After inactivating the Dpn I enzyme by heating at 80 °C for 30 min, the digested PCR products were transformed into *E. coli* XL1-Blue competent cells by the chemical transformation method [8]. The transformants were plated on LB agar supplemented with ampicillin (100μg/ml) and cultured overnight in a 37°C incubator.

***Expression of mutant α-amylases and preparation of crude enzymes***

The transformant colonies were inoculated using toothpicks into 96 well deep-well plates containing 400 µl liquid LB medium with ampicillin (100 μg/ml) per well and pre-cultured for 20 h at 220 rpm and 37°C on a vibrational shaker. The pre-cultures were divided into two equal quantities. One was further induced by the addition of IPTG (1 mM) and maintained for another 24 h at 220 rpm and 37°C on a vibrational shaker. The other was transferred into new 96 well deep-well plates and kept at 4°C for inoculation. The induced cells were harvested by centrifugation at 4,000 rpm for 20 min, and the pellets were washed once and suspended again in 200 μL per well 0.05 M NaH2PO4-citrate buffer pH 6.5 containing 0.1% Triton X-100. The suspension in the deep-well plates was heated at 65 °C for 30 min and the cell debris was removed by centrifugation at 4,000 rpm for 20 min. The supernatants constituted crude enzymes. To efficiently screen out the improved mutant α-amylases, the supernatants were subjected to another 10 min of heat inactivation at 65 °C and the precipitation was removed by centrifugation at 4,000 rpm for 20 min.

***Initial screening of CCSM library by starch-iodine method***

The enzymatic activity of crude enzymes after heat treat was assayed using the starch-iodine method [9]. A 10 μL volume of crude enzyme was added to 100 μL 0.5% soluble starch in 0.05 M NaH_2_PO_4_-citrate buffer pH 6.5 and the samples were incubated at 65°C for 5 min. Then 55 μL 0.1M HCL and 400 μL dilute iodine solution (40.2 mg/ml KI, 0.088 mg/ml I_2_) were added to show the residual starch by forming blue starch-iodine complex. For each CCSM library, 1001 mutants were randomly selected and assayed in eleven 96 well deep-well plates with two blank wells and three wells inoculated with wild-type strain in each plate as controls. The top 8 mutants from each plate were chosen as the potential hits, making 88 variants in total for each library.

***Rescreening of potential hits by DNS***

To distinguish true hits from false positives, the potential hits from the first screening were rescreened by comparing their specific activities of crude enzymes after heat treatment using the DNS method [10]. The protein concentration of crude enzyme was measured using the Bradford method with BSA as a standard [11]. All the measures were carried out in triplicate using a microplate reader (Infinite 200, Tecan, Austria). The relative activity of each potential hit was normalized by dividing the specific activity of each hit by that of the wild-type control from the same plate. The top 12 mutants, with the best relative activities, were defined as hits.

The chosen hits were further characterized by their relative activities and half inactivation temperature ($T_{50}^{30}$) using the freshly prepared crude enzymes released by the ultrasonic [8]. The $T_{50}^{30}$ value is the temperature at which the enzyme loses 50% of its original activity after 30 min heat treatment. The improved mutants that showed outstanding relative activities were sequenced to confirm by Shanghai DNA Biotechnologies (Shanghai, China).

***Purification and characterization of α-amylase***

The confirmed mutant α-amylase gene was expressed in *E.coli* JM109 and the recombinant α-amylase was purified using Ni-NTA metal-chelate affinity chromatography (Ni-NTA, Qiagen, Hilden, Germany). The purified protein was desalted using ultrafiltration membrane (cut-off molecule weight of 10,000 Dalton) (Millipore, U.S.). The homogeneity of purified protein was monitored by SDS-PAGE. The protein concentration was determined using the Bradford method [11]. The enzymatic parameters of α-amylases were deduced from the Michaelis–Menten plots.

The activity of α-amylase was determined in a reaction mixture comprising 50 mM Na2HPO4-citrate acid buffer (pH 6.5) containing 0.5% soluble starch and properly diluted purified α-amylase (containing up to 2 µg/mL). The mixture was incubated at 65°C for 5 min, and the amount of sugar released was determined with 3,5-dinitrosalicylic acid (DNS) [10]. One unit (U) of α-amylase was here defined as the amount of enzyme that could catalyze the release of 1 µmol of glucose equivalent per minute in 50  mM Na_2_HPO4-citrate acid buffer (pH 6.5) at 65°C. All assays were performed in triplicate.

**Reference**

1. Gouveia-Oliveira R, Pedersen AG: **Finding coevolving amino acid residues using row and column weighting of mutual information and multi-dimensional amino acid representation**. *Algorithms Mol Biol* 2007, **2**:12.

2. Gouveia-Oliveira R, Roque FS, Wernersson R, Sicheritz-Ponten T, Sackett PW, Molgaard A, Pedersen AG: **InterMap3D: predicting and visualizing co-evolving protein residues**. *Bioinformatics* 2009, **25**(15):1963-1965.

3. **The InterMap3D 1.3 Server**[<http://www.cbs.dtu.dk/services/InterMap3D/>]

4. Guex N, Peitsch MC: **SWISS-MODEL and the Swiss-PdbViewer: an environment for comparative protein modeling**. *Electrophoresis* 1997, **18**(15):2714-2723.

5. **Swiss-Model protein modeling server** [<http://swissmodel.expasy.org/>]

6. DeLano WL: **The PyMOL Molecular Graphics System** (2002) DeLano Scientific, Palo Alto, CA, U.S. [<http://www.pymol.org>].

7. Hogrefe HH, Cline J, Youngblood GL, Allen RM: **Creating randomized amino acid libraries with the QuikChange Multi Site-Directed Mutagenesis Kit**. *Biotechniques* 2002, **33**(5):1158-1160, 1162, 1164-1155.

8. Sambrook J, Russell D: **Molecular Cloning: A Laboratory Manual** third edn. New York: Cold Spring Harbor Laboratory Press; 2001.

9. Fuwa H: **A new method for microdetermination of amylase activity by the use of amylose as the substrate**. *J Biochem* 1954, **41**(5):583-603.

10. Miller GL: **Use of dinitrosalicylic acid reagent for determination of reducing sugar**. *Anal Chem* 1959, **31**(3):426-428.

11. Bradford MM: **A rapid and sensitive method for the quantitation of microgram quantities of protein utilizing the principle of protein-dye binding**. *Anal Biochem* 1976, **72**:248-254.
