# Supplementary material for: Improving the thermostability of alpha-amylase by combinatorial coevolving-site saturation mutagenesis
Source: BMC Bioinformatics. 2012 Oct 11;13:263. doi: 10.1186/1471-2105-13-263 (PMC3478181; doi:10.1186/1471-2105-13-263)
Supplement: Additional file 2 — Table SA2. Information on sequences homologous to Amy7C identified in Uniprot. This file provides the information including Accession number, name, origin strain and number of amino acids on the sequences analogous to Amy7C identified in Uniprot and employed to find the coevolving sites through the InterMap3D server in this study. [file 1471-2105-13-263-S2.doc]

Table A2 Information on sequences homologous to Amy7C identified in Uniprot

| Identifier | Code in UniprotKB | Origin | Length (AA) | Protein |
| --- | --- | --- | --- | --- |
| H9B4I9_BACIU | H9B419 | *Bacillus subtilis* | 424 | Amy7D |
| O82953_BACSU | O82953 | *Bacillus subtilis* | 659 | Alpha-amylase |
| AMY_BACSU | P00691 | *Bacillus subtilis* | 659 | AmyE |
| Q6PMJ3_BACSU | Q6PMJ3 | *Bacillus subtilis* | 659 | Alpha-amylase |
| Q9R9H7_BACSU | Q9R9H7 | *Bacillus subtilis* | 659 | Alpha-amylase |
| Q45520_BACSU | Q45520 | *Bacillus subtilis* | 477 | Alpha-amylase |
| Q6U833_BACSU | Q6U833 | *Bacillus subtilis* | 649 | Alpha-amylase |
| Q45N23_BACSU | Q45N23 | *Bacillus subtilis* | 659 | Alpha-amylase |
| Q45516_BACSU | Q45516 | *Bacillus subtilis* | 477 | Alpha-amylase |
| Q4QZ39_BACSU | Q4QZ39 | *Bacilus subtilis* | 465 | Amylase |
| O85007_9LACO | O85007 | *Lactobacillus manihotivorans* | 479 | Alpha-amylase |
| O50582_STRBO | O50582 | ***Streptococcus bovis*** | 742 | Alpha-amylase |
| Q5JB42_BIFAD | Q5JB42 | [*Bifidobacterium adolescentis*](http://www.uniprot.org/taxonomy/1680) | 596 | Alpha-amylase |
| A6LR71_CLOB8 | A6LR71 | ***Clostridium beijerinckii*** | 666 | Alpha-amylase |
| AMY_CLOAB | P23671 | [*Clostridium acetobutylicum*](http://www.uniprot.org/taxonomy/272562) | 760 | Alpha-amylase |
| Q97TK3_CLOAB | **Q97TK3** | ***Clostridium acetobutylicum*** | 561 | Alpha-amylase |
