# Supplementary material for: Improving the thermostability of alpha-amylase by combinatorial coevolving-site saturation mutagenesis
Source: BMC Bioinformatics. 2012 Oct 11;13:263. doi: 10.1186/1471-2105-13-263 (PMC3478181; doi:10.1186/1471-2105-13-263)
Supplement: Additional file 3 — Table SA3. Multiple Sequence Alignment in CLUSTAL format obtained by MAFFT method in this study. This file provides the Multiple Sequence Alignment in CLUSTAL format of the sequences homologous to Amy7C obtained by MAFFT method to find the coevolving sites through InterMap3D server in this study, [file 1471-2105-13-263-S3.docx]

Table A3 Multiple Sequence Alignment in CLUSTAL format obtained by MAFFT method in this study

H9B4I9_BACIU M-----------------------------------------------------------

O82953_BACSU M-------FEKR--FKTSLLPL-----FAGFLLLFHLVLSGPAAANAETANKSNKVT---

AMY_BACSU M-------FAKR--FKTSLLPL-----FAGFLLLFHLVLAGPAAASAETANKSNELT---

Q6PMJ3_BACSU M-------FAKR--FKTSLLPL-----FAGFLLLFHLVLAGPAAASAETANKSNELT---

Q9R9H7_BACSU M-------FAKR--FKTSLLPL-----FAGFLLLFHLVLAGPAAASAETANKSNELT---

Q45520_BACSU M-------FAKR--FKTSLLPL-----FAGFLLLFYLVLAGPAAASAETANKSNELT---

Q6U833_BACSU M-------FAKR--FKTSLLPL-----FAGFLLLFYLVLAGPAAASAETANKSIELT---

Q45N23_BACSU M-------FAKR--FKTSLLPL-----FAGFLLLFHLVLAGPNAANAETANKSNELT---

Q45516_BACSU M-------FAKR--FKTSLLPL-----FAGFLLLFYLVLAGPAAASAETANKSNELT---

Q4QZ39_BACSU M-------FAKR--FKTSLLPL-----FAGFLLLFHLVLAGPAAASAETANKSNELT---

O85007_9LACO M-------KKKKSFWLVSFLVIVASVFFISFGLSNHSKQVVQADSDTTSTDDSSNDT---

O50582_STRBO MTFQNKVNLKKK--MKKSLGSL--------------LILTAIGAGGLVQVKVVNADE---

Q5JB42_BIFAD MKHRKPAPAWHRLGLKISKKVV---VGITAAATAFGGLAIASTAAQASTDRDSYADTVEN

A6LR71_CLOB8 MRKKIISRLIAS-GLFLSLNLL--------PISIPS---FGTNGILTTAYAASSTSL---

AMY_CLOAB MSKR--SKLLKR-RM-LSLSVICVLIGY-GPVFNPV---RSQAKVMT--YSSENREL---

Q97TK3_CLOAB M-KRIFS--LRK-SL---LAVVVTLTAFSGSLFYPISNRFQNVQAVTYNVQPVTSNL---

*

H9B4I9_BACIU -------------SSVKNGTILHAWNWSFNTLTQNMKEIRDAGYAAIQTSPINQVKEGNQ

O82953_BACSU ------------ASSVKNGTILHAWNWSFNTLTQNMKDIRDAGYAAIQTSPINQVKEGNQ

AMY_BACSU ------------APSIKSGTILHAWNWSFNTLKHNMKDIHDAGYTAIQTSPINQVKEGNQ

Q6PMJ3_BACSU ------------APSIKSGTILHAWNWSFNTLKHNMKDIHDAGYTAIQTSPINQVKEGNQ

Q9R9H7_BACSU ------------APSIKSGTILHAWNWSFNTLKHNMKDIHDAGYTAIQTSPINQVKEGNQ

Q45520_BACSU ------------APSIKSGTILHAWNWSFNTLKHNMKDIHDAGYTAIQTSPINQVKEGNQ

Q6U833_BACSU ------------APSIKSGTILHAWNWSFNTLKHNMKDIHDAGYTAIQTSPINQVKEGNQ

Q45N23_BACSU ------------APSIKSGTILHAWNWSFNTLKHNMKDIHDAGYTAIQTSPINQVKEGNQ

Q45516_BACSU ------------APSIKSGTILHAWNWSFNTLKHNMKDIHDAGYTAIQTSPINQVKEGNQ

Q4QZ39_BACSU ------------APSIKSGTILHAWNWSFNTLKHNMKDIHDAGYTAIQTSPINQVKEGNQ

O85007_9LACO ------------ADSVSDGVILHAWCWSFNTIKNNLKQIHDAGYTTVQTSPVNEVKVGNS

O50582_STRBO ------------QVSMKDGTILHAWCWSFNTIKDNMQAIKDAGYTSVQTSPINTVVAGEG

Q5JB42_BIFAD TTFEQARKHYGLAQQMSEGATLHAWEWSFKTIEENIPAIAEAGYTSVQTEPISAIHNGGK

A6LR71_CLOB8 ------------PSNAKDGAILHAFDWSFATIKNELPNIAAAGYKSVQVSPVQGTK---S

AMY_CLOAB ------------PENTKDGVMLHAFDWSFNNIKKELPSIAAAGYKAVQVSPVQGTK---S

Q97TK3_CLOAB ------------PAKTEDGVIAQAFTWRFKDIQDHLQEFKDDGYKAILVSPVQRTP---K

. ..*. :*: * * : ..: : ** :: ..*:.

H9B4I9_BACIU GDKSMSNWYWLYQPTSYQIGNRYLGTEQEFKDMCAAAEKYGVKVIVDAVVNHTTSD----

O82953_BACSU GDKSMSNWYWLYQPTSYQIGNRYLGTEQEFKDMCAAAEKYGVKVIVDAVVNHTTSD----

AMY_BACSU GDKSMSNWYWLYQPTSYQIGNRYLGTEQEFKEMCAAAEEYGIKVIVDAVINHTTSD----

Q6PMJ3_BACSU GDKSMSNWYWLYQPTSYQIGNRYLGTEQEFKEMCAAAEEYGIKVIVDAVINHTTSD----

Q9R9H7_BACSU GDKSMSNWYWLYQPTSYQIGNRYLGTEQEFKEMCAAAEEYGIKVIVDAVINHTTSD----

Q45520_BACSU GDKSMSNWYWLYQPTSYQIGNRYLGTEQEFKEMCAAAEEYGIKVIVDAVINHTTSD----

Q6U833_BACSU GDKSMSNWYWLYQPTSYQIGNRYLGTEQEFKEMCAAAEEYGIKVIVDAVINHTTSD----

Q45N23_BACSU GNKSMLNWYWLYQPTSYQIGNRYLGTEQEFKEMCAAAEEYGIKVIVDAVINHTTSD----

Q45516_BACSU GDKSMSNWYWLYQPTSYQIGNRYLGTEQEFKEMCAAAEEYGIKVIVDAVINHTTFD----

Q4QZ39_BACSU GNKSMSNWYWLYQPTSYQIGNRYLGTEQEFKEMCAAAEEYGIKVIVDAVINHTTSD----

O85007_9LACO ASKSLNNWYWLYQPTKYSIGNYYLGTEAEFKSMCAAAKEYNIRIIVEATLNDTTSD----

O50582_STRBO GNKSLKNWYYQYQPTIYKIGNYQLGTEEEFKEMNRVADQYGIKIIVDAVLNHTTSD----

Q5JB42_BIFAD GMIFTENWYYVYQPTDTTIGNWVMGTEDDLKSLCNAAHKYGVRIIVDVVANHMTAT----

A6LR71_CLOB8 SSKDPSQWWLLYQPTNQSVGNAQLGNYDDFKALCTEADKYGISIVVDVVMNHMANNGNPD

AMY_CLOAB NSTNSSDWWLLYQPTNQAIGNAQLGSYDDFKSLCSEAKNYGISIVVDVVMNHMANNGNDD

Q97TK3_CLOAB ----AGDWWLLYQPCNFHIGNAQLGTYDDFKNLCSAANQYGIKIMVDALLNHVATS-SPG

:*: *** :** :*. ::* : *.:*.: ::*:. *. :

H9B4I9_BACIU -------YGAISDEIKRIPNW------TH--GNTQIKNWSDRWDITQNALLGLYDWNTQN

O82953_BACSU -------YGAISDEIKRIPNW------TH--GNTQIKNWSDRWDITQNALLGLYDWNTQN

AMY_BACSU -------YAAISNEVKSIPNW------TH--GNTQIKNWSDRWDVTQNSLLGLYDWNTQN

Q6PMJ3_BACSU -------YAAISNEVKSIPNW------TH--GNTQIKNWSDRWDVTQNSLLGLYDWNTQN

Q9R9H7_BACSU -------YAAISNEIKSIPNW------TH--GNTQIKNWSDRWDVTQNSLLGLYDWNTQN

Q45520_BACSU -------YAAISNEVKSIPNW------TH--GNTQIKNWSDRWDVTQNSLLGLYDWNTQN

Q6U833_BACSU -------YAAISNEVKSIPNW------TH--GNTQIKNWSDRWDVTQNSLLGLYDWNTQN

Q45N23_BACSU -------YAAISNEIKSIPNW------TH--GNTQIKNWSDRWDVTQNSLLGLYDWNTQN

Q45516_BACSU -------YAAISNEVKSIPNW------TH--GNTQIKNWSDRWDVTQNSLLGLYDWNTQN

Q4QZ39_BACSU -------YAAISNEIKSIPNW------TH--GNTQIKNWSDRWDVTQNSLLGLYDWNTQN

O85007_9LACO -------YRQFSVEIKSISNW------TH--GNTQISNWSIREDVTQNSLLGLYGLNAQN

O50582_STRBO -------YNQISQEIKNIPNW------TH--GNTLISDWHNRYDVTQNALLTLYDWNTQN

Q5JB42_BIFAD -------WGAIADRWKKSEYY------HHDCNDGDVQDWNNRYQVTHCKLLGLYDINTEN

A6LR71_CLOB8 Q-----LDSSIDPSFKD-PNL------YH--NQGQCSNWTNRYDVTQKGI-GMPDLNTQN

AMY_CLOAB E-----VASEVDPSFKD-PSL------YH--HNGQCTDWNNRQDVTQEGI-GMPDLNTQS

Q97TK3_CLOAB Q-----WDNSVDDSLKH-REL------YH--NQGSCNDYKDRYQVTQKDIGGLLDLATQR

. * * : :: * ::*: : : . ::

H9B4I9_BACIU TEVQAYLKGFLERALNDGADGFRYDAAKHIELPD--DG--NYGSQFWPNIT----NTSAE

O82953_BACSU TEVQAYLKGFLERALNDGADGFRYDAAKHIELPD--DG--NYGSQFWPNIT----NTSAE

AMY_BACSU TQVQSYLKRFLDRALNDGADGFRFDAAKHIELPD--DG--SYGSQFWPNIT----NTSAE

Q6PMJ3_BACSU TQVQSYLKRFLERALNDGADGFRFDAAKHIELPD--DG--SYGSQFWPNIT----NTSAE

Q9R9H7_BACSU TQVQSYLKRFLERALNDGADGFRFDAAKHIELPD--DG--SYGSQFWPTIT----NTSAE

Q45520_BACSU TQVQSYLKRFLERALNDGADGFRFDAAKHIELPD--DG--SYGSQFWPNIT----NTSAE

Q6U833_BACSU TQVQSYLKRFLDRALNDGADGFRFDAAKHIELPD--DG--SYGSQFWPNIT----NTSAE

Q45N23_BACSU TQVQSYLKRFLERALNDGADGFRFDAAKHIELPD--DG--SYGSQFWPNIT----NTSAE

Q45516_BACSU TQVQSYLKRFLERALNDGADGFRFDAAKHIELPD--DG--SYGSQFWPNIT----NTSAE

Q4QZ39_BACSU TQVQSYLKRFLERALNDGADGFRFDAAKHIELPD--DG--SYGSQFWPNIT----NTAAE

O85007_9LACO SQVQTYLKNYLERLISDGASGFRYDAAKHIELPSQYDG--SYGSNFWPNIT----DNGSE

O50582_STRBO EYVQQYLLSYLKQAVADGADGFRYDAAKHIELP----G--EYGSNFWNVIL----NNGSE

Q5JB42_BIFAD TKTANMMHDFLVQAVNDGVDGFRFDAAKHIELPDEYN-----GSQYWNIIL----NNGAQ

A6LR71_CLOB8 STVQNKAITFLNQCIDAGADGFRFDAAKHIETNIGLDSNQSWSGNYWSNVLGNLHNKSNL

AMY_CLOAB SAVQSKAITFLNQCVDAGATGFRFDAAKHIETDLGLDANKSWSGNYWENVLGSLHNKSNL

Q97TK3_CLOAB TDVQDMEIQFLNECIDAGAGGFRFDSAKHIETNSGEDSGKPWASDYWGRVLSSLHNRNNL

. :* . : *. ***:*:***** ..::* : :

H9B4I9_BACIU FQYGEILQD-SAS-RDTAYANYMNVTASN-------YGHSIRSALKNRN-LSVSNISHYA

O82953_BACSU FQYGEILQD-SAS-RDTAYANYMNVTASN-------YGHSIRSALKNRI-LSVSNISHYA

AMY_BACSU FQYGEILQD-SAS-RDAAYANYMDVTASN-------YGHSIRSALKNRN-LGVSNISHYA

Q6PMJ3_BACSU FQYGEILQD-SAS-RDAAYANYMDVTASN-------YGHSIRSALKNRN-LGVSNISHYA

Q9R9H7_BACSU FQYGEILQD-SAS-RDAAYANYMDVTASN-------YGHSIRSALKNRN-LGVSNLSHYA

Q45520_BACSU FQYGEILQD-SAS-RDAAYANYMDVTASN-------YGHSIRSALKNRN-LGVSNISHYA

Q6U833_BACSU FQYGEILQD-SAS-RDAAYANYMDVTASN-------YGHSIRSALKNRN-LGVSNISHYA

Q45N23_BACSU FQYGEILQD-SAS-RDASYANYMNVTASN-------YGHSIRSALKNRN-LGVSNISHYA

Q45516_BACSU FQYGEILQD-SAS-RDAAYANYMDVTASN-------YGHSIRSALKNRN-LGVSNISHYA

Q4QZ39_BACSU FQYGEILQD-SAS-RDASYANYMNVTASN-------YGHSIRSALKNRN-LGVSNISHYA

O85007_9LACO FQYGEVLQD-SIS-KESDYANYMSVTASN-------YGNTIRNALKNRD-FTASTLQNFN

O50582_STRBO FQYGEILQD-DVS-NDAGYGKLMSITASN-------YGQKIRSALKDRH-ISAGNLMNYQ

Q5JB42_BIFAD FQYGEVLQD-SIS-RDSDYAKLFSSHSKNGGGVTDLYGSKLRGALNSKN-LNAGTLSDWS

A6LR71_CLOB8 FIYGEILQDGSVD-NIASYESFMNVTASN-------YGGAVRSAVTSTN-LS---SLGTT

AMY_CLOAB YIYGEVLQDGKVD-NISAYESFMNVEASA-------YDGSLRGAIKSGD-LT---N-AQG

Q97TK3_CLOAB YLYGEVLPDYG-D-NDEVYRSYFDITAES-------YGSTIRNAVQNKN-LNGLLNINFS

: ***:* * . . * . :. :. *. :*.*: . :

H9B4I9_BACIU SD-VSADKLVTWVESHDTYANDDEESTWMSDDDIRLGWAVIGSRSGSTPLFFSRPEG-GG

O82953_BACSU SD-VSADKLVTWVESHDTYANDDEESTWMSDDDIRLGWAVIGSRSGSTPLFFSRPEG-GG

AMY_BACSU SD-VSADKLVTWVESHDTYANDDEESTWMSDDDIRLGWAVIASRSGSTPLFFSRPEG-GG

Q6PMJ3_BACSU SD-VSADKLVTWVESHDTYANDDEESTWMSDDDIRLGWAVIASRSGSTPLFFSRPEG-GG

Q9R9H7_BACSU SD-VSADKLVTWVESHDTYANDDEESTWMSDDDIRLGWAVIASRSGSTPLFFSRPEG-GG

Q45520_BACSU SD-VSADKLVTWVESHDTYANDDEESTWMSDDDIRLGWAVIASRSGSTPLFFSRPEG-GG

Q6U833_BACSU SD-VSADKLVTWVESHDTYANDDEESTWMSDDDIRLGWAVIASRSGSTPLFFSRPEG-GG

Q45N23_BACSU SD-VPADKLVTWVESHDTYANDDEESTWMSDDDIRLGWAVIASRSGSTPLFFSRPEG-GG

Q45516_BACSU YD-VSADKLVTWVESHDTYANDDEESTWMSDDDIRLGWAVIASRSGSTPLFFSRPEG-GG

Q4QZ39_BACSU SD-VSADKLVTWVESHDTYANDDEESTWMSDDDIRLGWAVIASRSGSTPLFFSRPEG-GG

O85007_9LACO IS-VPASKLVTWVESHDNYANDDQVSTRMNSSDIKLGWAVVASRSGSVPLFFDRPVD-GG

O50582_STRBO VSGVDAANLVTWVESHDNYANDDQESTWMNDSDIGLGWAMITARAKGTPLFFSRPVG-GG

Q5JB42_BIFAD NS-ASPSNLVSWLESHDNYSNSDRESTGMSEWQMTMGWGVIGSRSQTMPLYFDRPVGSGG

A6LR71_CLOB8 LGGVDSSKAVDFVETHDTY--EDGSSKNLTDTQRKLGWAIAAARANATPLFFDRPTG---

AMY_CLOAB MGGLDSNKCVDMLETHDEY--EHNESKDLTDWQRKAGWAIAASRAGSVPLFFDRPTG---

Q97TK3_CLOAB DHNIPSSQALCYVESHDNY--EHNQSSSTSDWNIKMGFAILDARAQLTPQFFIRPS----

. : : :*:** * .. *. .. : *:.: :*: * :* **

H9B4I9_BACIU NGVRFPGKSQIGDRGSALFKDQAITAVNQFHNEMAGQ--PEELSNPNGNNQIFMNQRGSK

O82953_BACSU NGVRFPGKSQIGDRGSALFKDQAITAVNQFHNEMAGQ--PEELSNPNGNNQIFMNQRGSK

AMY_BACSU NGVRFPGKSQIGDRGSALFEDQAITAVNRFHNVMAGQ--PEELSNPNGNNQIFMNQRGSH

Q6PMJ3_BACSU NGVRFPGKSQIGDRGSALFEDQAITAVNRFHNVMAGQ--PEELSNPNGNNQIFMNQRGSH

Q9R9H7_BACSU NGVRFPGKSQIGDRGSALFEDQAITAVNRFHNVMAGQ--PEELSNPNGNNQIFMNQRGSH

Q45520_BACSU NGVRFPGKSQIGDRGSALFEDQAITAVNRFHNVMAGQ--HEELSNPNGNNQIFMNQRGSH

Q6U833_BACSU NGVRFPGKSQIGDRGSALFEDQAITAVNRFHNVMAGQ--PEELSNPNGNNQIFMNQRISH

Q45N23_BACSU NGVRFPGKSQIGDRGSALFEDQAITAVNRFHNVMAGQ--PEELSNPNGNNQIFMNQRGSH

Q45516_BACSU NGVRFPGKSQIGDRGSALFEDQSITAVNRFHNVMAGQ--PEELSNPNGNNQIFMNQRGSH

Q4QZ39_BACSU NGVRFPGKSQIGDRGSALFEDQANTAVNRFHNVMAGQ--PEELSNPNGNNQIFMNQRGSH

O85007_9LACO NGTRFPGSSEIGDAGSSLYYDKAVVAVNKFHNAMAGQ--SEYISNPNGNTKIFENERGSK

O50582_STRBO NGTRFPGQSQIGDAGSNLYKDATVTAVNKFHNAMVGE--SEYLRNPGGDEQVAMIERGTK

Q5JB42_BIFAD SQPQFAEKSKLGDAGADSWKDAQVVAVNHFRNTMNNNKASEYLRN-CGANSCLMVERYIK

A6LR71_CLOB8 ---------NIGSKGDDLWKDADIVAINNFHNAMVGK--NEYIRW-TNNNTTMLIDRGTD

AMY_CLOAB ---------NIGSEGDALWKDSDVVAVNEFHNAMAGQ--NEYLRL-QNNNKAMIIERGSK

Q97TK3_CLOAB ---------------DNSWKDSDIAAVNKFHNAMAGQ--SEYLR--NPRNETIMIERGNQ

: * .*:*.*:* * .: * : :* .

H9B4I9_BACIU -------GVVLANAGSSSVTINTSTKLPDGRYDNRAGAG-SFQVANGKLT-GTINARSAA

O82953_BACSU -------GVVLANAGSSSVTINTSTKLPDGRYDNRAGAG-SFQVANGKLT-GTINARSAA

AMY_BACSU -------GVVLANAGSSSVSINTATKLPDGRYDNKAGAG-SFQVNDGKLT-GTINARSVA

Q6PMJ3_BACSU -------GVVLANAGSSSVSINTPTKLPDGRYDNKAGAG-SFQVNDGKLT-GTINARSVA

Q9R9H7_BACSU -------GVVLANAGSSSVSINTATKLPDGRYDNKAGAG-SFQVNDGKLT-GTINARSVA

Q45520_BACSU -------GVVLANAGSSSVSINTATKLPDGRYDNKAGAG-SFQVNDGKLT-GTINARSVA

Q6U833_BACSU -------GVVLANAGSSSVSINTATKLPDGRYDNKAGAG-SFQVNDGKLT-GTINARSVA

Q45N23_BACSU -------GVVLANAGSSSVSINTPTKLPDGRYDNKAGAG-SFQVNDGKLT-GTINARSVA

Q45516_BACSU -------GVVLANAGSSSVSINTPTKLPDGRYDNKAGAG-SFQVNDGKLT-GTINARSVA

Q4QZ39_BACSU -------GVVLANAGSSSVSINTPTKLPDGRYDNKAGAG-SFQVNDGKLT-GTINARSVA

O85007_9LACO -------GVVFANASDSSYSLNVKTSLADGTYENKAGSD-EFTVKNGYLT-GTIQGREVV

O50582_STRBO -------GAVIVNLVDGDKQINSETNLADGTYTDKVSGR-QFNVSNGRIT-GSVPSRSAV

Q5JB42_BIFAD DGNFKNDGVTITNMGDTQELSGTATNLDDGTYKDQVSGG-TITVSGGKITSGSAPGGKIS

A6LR71_CLOB8 -------GTVIVNDGG-STSINSPTNLANGTYTNKGSANCTLTVSNGTIS-GNIPANSVI

AMY_CLOAB -------GAVIVNEGD-SFNLNTPTNLEDGNYDNHGSATDSLTVSQGRMT-GTVPANSII

Q97TK3_CLOAB -------GMVIVNVGG-DTPIDSATNLKDGSYTNKASANCTLNVSNGRIT-GNIPGGQII

* .:.* . . . *.* :* * :: .. : * * :: *. . .

H9B4I9_BACIU VLYP--------------------------------------------------------

O82953_BACSU VLYP--------------------------------------------------------

AMY_BACSU VLYP--------------------------------------------------------

Q6PMJ3_BACSU VLYP--------------------------------------------------------

Q9R9H7_BACSU VLYP--------------------------------------------------------

Q45520_BACSU VLYP--------------------------------------------------------

Q6U833_BACSU VLYP--------------------------------------------------------

Q45N23_BACSU VLYP--------------------------------------------------------

Q45516_BACSU VLYP--------------------------------------------------------

Q4QZ39_BACSU VLYP--------------------------------------------------------

O85007_9LACO VLYG--------------------------------------------------------

O50582_STRBO VLYD--------------------------------------------------------

Q5JB42_BIFAD VFFT--------------------------------------------------------

A6LR71_CLOB8 VLYN----------------------------------------------DG--------

AMY_CLOAB VIYNKNSNPGSDRVTLSEQAAKAGDSVTITYDAGTTALKDASNVNLYWGYDGFSAATSKA

Q97TK3_CLOAB VLYN--------------------------------------------------------

*::

H9B4I9_BACIU ------------------------------------------------------------

O82953_BACSU ------------------------------------------------------------

AMY_BACSU ------------------------------------------------------------

Q6PMJ3_BACSU ------------------------------------------------------------

Q9R9H7_BACSU ------------------------------------------------------------

Q45520_BACSU ------------------------------------------------------------

Q6U833_BACSU ------------------------------------------------------------

Q45N23_BACSU ------------------------------------------------------------

Q45516_BACSU ------------------------------------------------------------

Q4QZ39_BACSU ------------------------------------------------------------

O85007_9LACO ------------------------------------------------------------

O50582_STRBO ------------------------------------------------------------

Q5JB42_BIFAD ------------------------------------------------------------

A6LR71_CLOB8 -----------SILTP---------------------------PVPSTYAPHSGYKV-DY

AMY_CLOAB MTSLGDNKWQTTITVPKEVTKNVNFSFTDGTSWDNNNGANWNIPLASNYLPHAGYKV-DY

Q97TK3_CLOAB -------------NVP---------------------------------TPNPGNDVIKV

H9B4I9_BACIU D-----------------------------------------------------------

O82953_BACSU DDIGNAPHVFLENYQTEAVHSFNDQLTVTLRANAKTTKAVYQINNGQETAFKDGDRLTIG

AMY_BACSU DDIAKAPHVFLENYKTGVTHSFNDQLTITLRADANTTKAVYQINNGPDDRRLRMEINSQS

Q6PMJ3_BACSU DDIAQAPHVFLENYKTGVTHSFNDQLTITLRADANTTKAVYQINNGPETAFKDGDQFTIG

Q9R9H7_BACSU DDIAKAPHVFLENYKTGVTHSFNDQLTITLRADANTTKAVYQINNGPETAFKDGDQFTIG

Q45520_BACSU DDIEIRCNTFFQ------------------------------------------------

Q6U833_BACSU DDIAKAPHVFLENYKTGVTHSFNDQLTITLRADANTFIKSIMDQIN-R-RRLRMEINSQS

Q45N23_BACSU DDIAKAPHVFLENYKTGVTHSFNDQLTITMRADAKTTKAVYQINNGPETAFKDGDQFTIG

Q45516_BACSU DDIEIRCNTFFQ------------------------------------------------

Q4QZ39_BACSU ------------------------------------------------------------

O85007_9LACO DPTSS-------------------------------------------------------

O50582_STRBO DQASQAAQVSVDGYKEG-DNSISKATEVTLKAK-NADSATYKLGNGQEVAYKDGDKVTVG

Q5JB42_BIFAD DNSGSVSATPGD------SSFKTDTTTVTLNAN-NVTDATYTTSEGKSGSYQDGDTITIG

A6LR71_CLOB8 DSSTLLQGNSFTLYYSGS---LANSSSVKLHWGYNGFLNPSDVTMTKGSDGFWAATIKI-

AMY_CLOAB DSSNLVSGNNFTIYYNGN---LANSSNVSLHWGVNGWSNMQNLAMVKDSNGFWEATIAI-

Q97TK3_CLOAB NPQSPVPGQNITVTYNAAARVLQNSSLIKMHWGYDSWKGTKDTSMTSLGNNLWQATVTV-

H9B4I9_BACIU ------------------------------------------------------HHHHH-

O82953_BACSU KED-PIGTTYNVKLTGTNGEGASRTQEYTFVKKDPSQTNIIGYQNPDHWGNVNAYIYKHD

AMY_BACSU EKEIQFGKTYTIMLKGTNSDGVTRTEKYSFVKRDPASAKTIGYQNPNHWSQVNAYIYKHD

Q6PMJ3_BACSU KGD-PFGKTYTIMLKGTNSDGVTRTEEYSFIKRDPASAKTIGYQNPNHWSQVNAYIYKHD

Q9R9H7_BACSU KGD-PFGKTYTIMLKGTNSDGVTRAEEYSFVKRDPASAKTIGYQNPNHWSQVNAYIYKHD

Q45520_BACSU ------------------------------------------------------------

Q6U833_BACSU EKEIQFGKTYTIMLKGTNSDGVTR-EKYSLPKRDPASAKTIGYQNPNHWSQVNAYIYKHD

Q45N23_BACSU KGD-PFGKTYTIMLKGTNSDGVTRTEEYSFIKRDPASAKTIGYQNPNHWSQVNAYIYKHD

Q45516_BACSU ------------------------------------------------------------

Q4QZ39_BACSU ------------------------------------------------------------

O85007_9LACO ------------------------------------------------------------

O50582_STRBO EGL-EAGQSTTLTLTATGADGQSTTKTYTFTMKDPSAETNIYFQNPDNWSEVYAYMYS--

Q5JB42_BIFAD AST-AIGDTITVKLQGKDADGQTVSATYKYTKKDP-------------------------

A6LR71_CLOB8 ----P-SSATKLDFDFTNGSNWDNNSSKDWHLQVSSSSVPVQVNPAPTASKTTTIYYNGN

AMY_CLOAB ----P-ASSNTLNFCFTNGSSWDNNNNNNWTLNTWSSVPKVQVTPAPEACKQISVYYNGS

Q97TK3_CLOAB ----PYEASSALNMVFTNGSTWDNNNNQNWEF----------------------------

H9B4I9_BACIU ------------------------------------------------------------

O82953_BACSU GGGAIELTGSWPG---------KAMTKNADGIYTLTLPANADTADAKVIFNNGSAQVPGQ

AMY_BACSU GSRVIELTGSWPG---------KPMTKNADGIYTLTLPADTDTTNAKVIFNNGSAQVPGQ

Q6PMJ3_BACSU GGQAIELTGSWPG---------KPMTKNADGIYTLTLPADTDTTNAKVIFNNGSAQVPGQ

Q9R9H7_BACSU GGRAIELTGSWPG---------KPMTKNADGIYTLTLPADTDTTNAKVIFNNGSAQVPGQ

Q45520_BACSU ------------------------------------------------------------

Q6U833_BACSU GSREIELTGSWPG---------KPMTKNADGIYTLTLPADTDTTNAKVIFNNGYAQVPGQ

Q45N23_BACSU GGQAIELTGSWPG---------KPMTKNADGIYTLTLPADTDTTNAKVIFNNGSAQVPGQ

Q45516_BACSU ------------------------------------------------------------

Q4QZ39_BACSU ------------------------------------------------------------

O85007_9LACO ------------------------------------------------------------

O50582_STRBO -AKDNKLLGAWPG---------TKMTKEASGRYSITVPASYAEEGVKVIFTNNQGSQYPQ

Q5JB42_BIFAD ------------------------------------------------------------

A6LR71_CLOB8 LAANSTSVILHWGYNDFTNPTDVTMTKQSDGRWAATITIPSATYSLNMAFKNDSGSWDSN

AMY_CLOAB LASSASNITLHWGCNGFTSPQDINMVKQADGRWLANITLPSGCYNVNMAFKDQSGTWDNN

Q97TK3_CLOAB ------------------------------------------------------------

H9B4I9_BACIU ------------------------------------------------------------

O82953_BACSU NHPGFDYVQNGLYNNSGLNGYLP-------------------------------------

AMY_BACSU NQPGFDYVLNGLYNDSGLSGSLP-------------------------------------

Q6PMJ3_BACSU NQPGFDYVQNGLYNDSGLSGSLP-------------------------------------

Q9R9H7_BACSU NQPGFDYVQNGLYNDSGLSGSLP-------------------------------------

Q45520_BACSU ------------------------------------------------------------

Q6U833_BACSU NQPGFDYVLNGLY-----------------------------------------------

Q45N23_BACSU NQPGFDYVQNGLYNDSGLSGSLP-------------------------------------

Q45516_BACSU ------------------------------------------------------------

Q4QZ39_BACSU ------------------------------------------------------------

O85007_9LACO ------------------------------------------------------------

O50582_STRBO NE-GFDFKAEGLYSKAGLMPDVPAGKTRVTFDNPGGWDSANAYLYYGNPVQYPLGVWPGT

Q5JB42_BIFAD ------------------------------------------------------------

A6LR71_CLOB8 SSANYNY-SISQ------------------------------------------------

AMY_CLOAB NSNNYNFSSTNN------------------------------------------------

Q97TK3_CLOAB --------NIAK------------------------------------------------

H9B4I9_BACIU -------------------------------------------------------H

O82953_BACSU -------------------------------------------------------H

AMY_BACSU -------------------------------------------------------H

Q6PMJ3_BACSU -------------------------------------------------------Y

Q9R9H7_BACSU -------------------------------------------------------H

Q45520_BACSU --------------------------------------------------------

Q6U833_BACSU --------------------------------------------------------

Q45N23_BACSU -------------------------------------------------------H

Q45516_BACSU --------------------------------------------------------

Q4QZ39_BACSU --------------------------------------------------------

O85007_9LACO --------------------------------------------------------

O50582_STRBO QMTKDDAGNFYLDLPEEYADVNAKIIFNQPGTSNQYPYSEGFNLVKSGNYNKDGLK

Q5JB42_BIFAD --------------------------------------------------------

A6LR71_CLOB8 --------------------------------------------------------

AMY_CLOAB --------------------------------------------------------

Q97TK3_CLOAB --------------------------------------------------------
