# Supplementary material for: Improving the thermostability of alpha-amylase by combinatorial coevolving-site saturation mutagenesis
Source: BMC Bioinformatics. 2012 Oct 11;13:263. doi: 10.1186/1471-2105-13-263 (PMC3478181; doi:10.1186/1471-2105-13-263)
Supplement: Additional file 4 — Table SA1. Plasmids, strains, and primers used in this study. This file includes the plasmids, host strains, and nucleotide primers used in this study. [file 1471-2105-13-263-S4.doc]

Table A1 Plasmids, strains, and primers used in this study

| Name | Description/ Primer sequence 5′-3′ | Source |
| --- | --- | --- |
| *E. coli* XL10-Gold | Genotype: endA1 glnV44 recA1 thi-1 gyrA96 relA1 lac Hte Δ(mcrA)183 Δ(mcrCB-hsdSMR-mrr)173 tet^R^ F'[proAB lacI^q^ZΔM15 Tn10(Tet^R^ Amy Cm^R^); clone and expression strain | Stratagene |
| *E.coli* JM109 | endA1 glnV44 thi-1 relA1 gyrA96 recA1 mcrB+ Δ(lac-proAB) e14- [F' traD36 proAB+ lacIq lacZΔM15] hsdR17(rK-mK+); expression strain | Promega |
| pSA7C | Recombinant plasmid pSE380 inserting coding sequence for wild-type α-amylase (Amy7D) with his-tag at the C-terminal; template plasmid | This lab |
| G89SM-S | 5′-GCCGCGGAAAAGTATNNKGTAAAAGTCATTGTCG-3′ | This work |
| G89SM-A | 5′-CGACAATGACTTTTACMNNATACTTTTCCGCGGC -3′ | This work  This work |
| D95SM-S | 5′-GTAAAAGTCATTGTCNNKGCGGTTGTCAATCATAC-3′ |
| D95SM-A | 5′-GTATGATTGACAACCGCMNNGACAATGACTTTTAC 3′ | This work  This work |
| H100SM-S | 5′-GATGCGGTTGTCAATNNKACCACCAGCGATTATG -3′ |
| H100SM-A | 5′-CATAATCGCTGGTGGTMNNATTGACAACCGCATC -3′ | This work  This work |
| D144SM-S | 5′-ATTGCTTGGGCTGTATNNKTGGAATACTCAGAATAC -3′ |
| D144SM-A | 5′-GTATTCTGAGTATTCCAMNNATACAGCCCAAGCAAT -3′ | This work  This work |
| T147SM-S | 5′-CTGTATGATTGGAATNNKCAGAATACTGAGGTG-3′ |
| T147SM-A | 5′-CACCTCAGTATTCTGMNNATTCCAATCATACAG-3′ | This work |
| N197SM-S | 5′-TGGCCGAATATCACANNKACATCGGCGGAGTTC -3′ | This work |
| N197SM-A | 5′-GAACTCCGCCGATGTMNNTGTGATATTCGGCCA -3′ | This work |

The primers are named after the amino acid sites at which the randomizations were performed. The underlined NNK indicates the degeneracy codon for the creation of the saturation mutagenesis library. The primer names were designated using the names of the mutated residues (single letter) + site numbering + SM (short for saturation mutagenesis) + “-” + primer orientation (S-sense primer, A-antisense primer).
