# Supplementary material for: Improving the thermostability of alpha-amylase by combinatorial coevolving-site saturation mutagenesis
Source: BMC Bioinformatics. 2012 Oct 11;13:263. doi: 10.1186/1471-2105-13-263 (PMC3478181; doi:10.1186/1471-2105-13-263)
Supplement: Additional file 5 — Figure SA1. Close-up views of residues at coevolving sites in Amy7C and its variants. This file depicts the position and interaction of the residues at coevolving sites in Amy7C (A), H100I (B), D95HT147S (C), H100MD144R (D), T147P (E), N197C (F), and G89FD95R (G). [file 1471-2105-13-263-S5.doc]

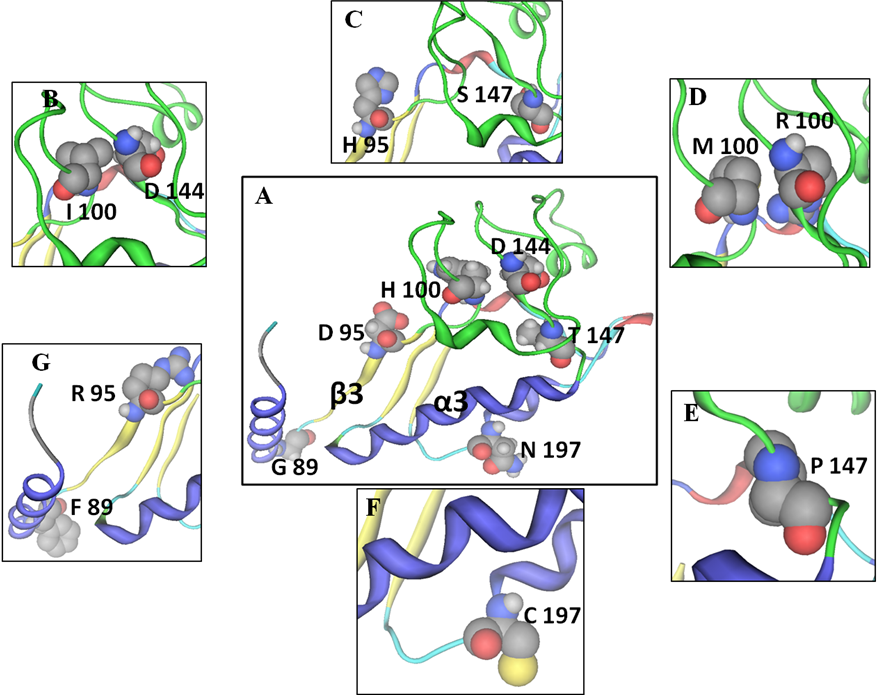


Figure A1 Close-up views showing the position and interaction of the residues at coevolving sites in Amy7C (A), H100I (B), D95HT147S (C), H100MD144R (D), T147P(E), N197C (F), and G89FD95R (G).
